# Ambrosia gall midges (Diptera: Cecidomyiidae) and their microbial symbionts as a neglected model of fungus-farming evolution

**DOI:** 10.1093/femsre/fuaf010

**Published:** 2025-04-02

**Authors:** Petr Pyszko, Hana Šigutová, Jan Ševčík, Michaela Drgová, Denisa Hařovská, Pavel Drozd

**Affiliations:** Department of Biology and Ecology, Faculty of Science, University of Ostrava, Chittussiho 10, 710 00 Ostrava, Czech Republic; Department of Biology and Ecology, Faculty of Science, University of Ostrava, Chittussiho 10, 710 00 Ostrava, Czech Republic; Department of Biology and Ecology, Faculty of Science, University of Ostrava, Chittussiho 10, 710 00 Ostrava, Czech Republic; Department of Biology and Ecology, Faculty of Science, University of Ostrava, Chittussiho 10, 710 00 Ostrava, Czech Republic; Department of Biology and Ecology, Faculty of Science, University of Ostrava, Chittussiho 10, 710 00 Ostrava, Czech Republic; Department of Biology and Ecology, Faculty of Science, University of Ostrava, Chittussiho 10, 710 00 Ostrava, Czech Republic

**Keywords:** ambrosia gall midges, fungal symbionts, fungus farming, phytomycetophagy, host specialization, host shift

## Abstract

Ambrosia gall midges (AGMs) represent an intriguing group within the Cecidomyiidae, one of the most diversified dipteran families. AGMs form galls on plants, where they cultivate and consume fungal symbionts (phytomycetophagy). This mutualistic relationship may play a critical role in larval nutrition, gall morphogenesis, and protection against natural enemies. Although most other fungus-farming taxa have been intensively studied, AGMs have largely been neglected. This review synthesizes current knowledge on the diversity, biology, and ecological interactions of AGM, highlighting the intricate relationships with their fungal symbionts. The implications for adaptive radiation and speciation are critically considered, including how fungal associations may have facilitated ecological flexibility and diversification. We also tackle the processes of coevolution, not only between AGM and their fungal symbionts but also involving plants and parasitoids. We identify the most pressing issues and discrepancies in the current understanding the AGM–fungi interactions. Key areas of future research should include elucidating fungal acquisition and transmission mechanisms, determining the specificity and diversity of AGM-associated fungal communities, understanding the evolutionary pathways leading to phytomycetophagy, and addressing taxonomic challenges within the AGM group, where species identification has been complicated by reliance on gall morphology and host specificity.

## Teaser

The intricate relationships between ambrosia gall midges and their fungal symbionts provide a compelling model for studying insect–fungus coevolution and mutualism, with significant implication for understanding insect speciation and host plant specialization.

## Introduction

Gall midges (Cecidomyiidae) represent an important and species-rich clade within the infraorder Bibionomorpha (*sensu* Ševčík et al. 2016) of the insect order Diptera. The family currently includes >6650 described species in 832 genera (Gagné and Jaschhof 2021). Nevertheless, the overall diversity is difficult to assess because new species are continually being described (Chimeno et al. 2022). Evidence is accumulating that Cecidomyiidae may be the most speciose dipteran family (Borkent et al. 2018, D’Souza et al. 2021), probably due to their narrow host plant specialization (Gagné 1989). According to an older estimate by Hebert et al. (2016), it may comprise ∼1.8 million species. They are currently classified in six subfamilies (Sikora et al. 2019, Gagné and Jaschhof 2021), of which the largest, higher gall midges (Cecidomyiinae), contains mostly taxa with herbivorous larvae forming galls in plants, but also fungivores and predators (Gagné and Jaschhof 2021). The remaining subfamilies, Catotrichinae, Lestremiinae, Micromyinae, Winnertziinae, and Porricondylinae, comprise only one-fourth of the described species, and their larvae are predominantly fungivorous or saproxylic (Gagné and Jaschhof 2021).

Among all feeding strategies, fungivorous Cecidomyiinae are understudied, and their biology is poorly known. Some of them, ambrosia gall midges (AGMs), have evolved a mixed feeding mode: phytomycetophagy. Therefore, they represent a unique evolutionary transition between mycophagy and herbivory (Docters van Leeuwen 1939, Simon et al. 2017). Although they are mostly host–plant specialists, they form galls where they may feed on the mycelium of cultivated fungal symbionts (Docters Van Leeuwen 1929). In insects, fungiculture has evolved independently at least six times (Dentinger and Bills 2018; Biedermann and Vega 2020), and has been intensively studied in woodwasps (Morgan 1968, Gilbertson 1984), bark and ambrosia beetles (Hulcr et al. 2020, Cole et al. 2021, Elkhateeb 2021, Strzałka et al. 2021, Diehl et al. 2023), fungus-growing termites (Aanen and Boomsma 2006), and leafcutter ants (De Fine Licht et al. 2013; reviewed by Mueller et al. 2005, Biedermann and Vega 2020). However, in AGM, insect–fungus mutualism was considered understudied 20 years ago (Sugiura et al. 2004), and this group remains understudied, even with the advent of advanced molecular techniques facilitating the study of hidden interactions.

**Figure 1.**
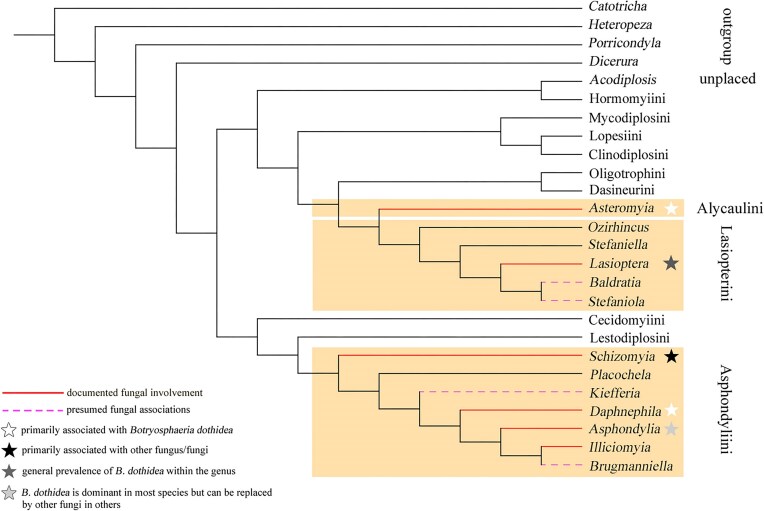
Illustrative cytochrome oxidase I (COI) gene-based cladogram depicting the hypothesized phylogenetic relationships among selected tribes of AGM, distinguished to the genus level, and other tribes of Cecidomyiidae, highlighting the principal fungal associations of AGM. Three focal tribes (indicated by background shading)—Alycaulini, Lasiopterini, and Asphondyliini—are hypothesized to represent the suspected origins of ambrosia-like feeding, with two independent evolutionary origins—one separate (Asphondyliini) and the other shared between two closely related tribes (Alycaulini and Lasiopterini). Line styles represent fungal symbioses: solid lines indicate documented fungal involvement, whereas dashed lines denote presumed fungal associations. Asterisk symbols indicate fungal specificity: open stars designate species primarily associated with *Botryosphaeria dothidea*, whereas filled stars indicate dominance of alternative fungal symbionts. In the genus *Lasioptera, B. dothidea* prevails in some species but is replaced by other fungi in others (hence the transition in background shading), whereas in the genus *Asphondylia, B. dothidea* is generally dominant but replaced by other fungi in certain species (represented by a paler shading). This continuum underscores the plasticity of fungal symbioses within AGM, with two possible evolutionary origins giving rise to varied degrees of host–fungus specificity.

AGMs have been proposed to belong to five tribes (Alycaulini, Lasiopterini, Asphondyliini, Cecidomyiini, and Oligotrophini) (Bissett and Borkent 1988, Roskam 1992, Yukawa and Rohfritsch 2005), although some of these assignments rely on limited or outdated evidence. However, the nutritive mycelia have not been adequately investigated in all tribes, and in Asphondyliini, Lasiopterini, and Alycaulini, they have only been partially studied. Therefore, the extent of their dependence on fungal symbionts remains unknown (Fig. 1).

In this review, we present a comprehensive synthesis of available information on the diversity of the AGM gall microbiome, its roles and functions, the specificity, and origin of AGM–fungi interaction, as well as a detailed review of the routes of symbiont transmission. We emphasize the role of fungi in the AGM host plant specialization and host shifts, and we also tackle the processes of coevolution, not only between AGM and their fungal symbionts, but also between fungi, plants, and parasitoids. We identify the most pressing issues and discrepancies in the current knowledge of the AGM–fungi interaction, and suggest a future direction for studies of this unique evolutionary association.

## Diversity of AGM gall microbiome and specificity of AGM–fungus interactions

Insect fungicultures typically comprise complex interactions between fungi and bacteria (Dentinger and Bills 2018; Li et al. 2021). It has even been proposed that endosymbiotic bacteria or their genes acquired by horizontal transfer may mediate gall induction in galling insects (Bartlett and Connor 2014, Giron and Glevarec 2014, Hammer et al. 2021). However, the bacterial microbiome of AGM galls has been completely neglected, except for a note mentioning presence of bacteria in mature galls (Rohfritsch 1992), and thus the complex interactions among bacteria and fungi, as well as their roles in the AGM galls, remain to be described.

Regarding fungal associates, not much is generally known about the extent of interdependence between individual AGM species and fungi. Obligate mutualism can be distinguished by specific adaptations in both partners (e.g. mycetangia in AGM, swollen fruiting bodies in fungi; see Biedermann and Vega 2020). In the case of AGM, these adaptations have been proven in above-mentioned tribes Alycaulini, Asphondyliini, and Lasiopterini (Table 1). Most species of Asphondyliini and Lasiopterini are associated with a specific symbiont: *Botryosphaeria dothidea* (Moug.) Ces. & de Not. (e.g. Bissett and Borkent 1988, Rohfritsch 2008, Adair et al. 2009, Kobune et al. 2012, Pan et al. 2015, Park et al. 2019, Pyszko et al. 2024; see Table 1). However, this fungus may also play a role in other AGM tribes (Heath and Stireman 2010, Janson et al. 2010, Kobune et al. 2012, Park et al. 2019), and may therefore have a long and important evolutionary association with AGM (Bissett and Borkent 1988, Veenstra et al. 2019). Apart from *B. dothidea* and its anamorphs (Slippers et al. 2004), such as *Macrophoma* (Bissett and Borkent 1988; see Tokuda 2012) or *Fusicoccum aesculi* (Pan et al. 2015, Zimowska et al. 2017, Bernardo et al. 2018), a wide variety of other fungi have been found in the galls of *Asphondylia* species. Records of particular fungal associations have been reported (Kobune et al. 2012, Rowan 2014), especially from the galls of *Lasioptera*, where fungi other than *B. dothidea* have been commonly found as primary symbionts (see Table 1). However, many of these reports need to be verified due to the existence of anamorphs and recent changes in fungal taxonomy.

**Table 1.** Overview of the most frequently reported AGM–fungi associations.^a^

Tribe	Species (AGM)	Groups of primary reported fungi	Modes of transmission	Spore-carrying structures	Benefit to fungi	Benefit to insect	Highlights of the interaction	Most important references
Alycaulini	*Asteromyia carbonifera*	*Botryosphaeria dothidea* ^b^, *Alternaria* spp.^b^, *Diaporthe* spp.^b^, *Phoma* ^b^, *Sclerotium asteris*	Ovipositing female; ^c^vertical (high level of specificity), ^c^horizontal (*B. dothidea* genetically identical to free-living populations, molecular evidence)	Yes	Proliferation, dispersal	Nutrition, protection from parasitoids; possible parasitism on fungus	Larva can grow on *B. dothidea* alone, *B. dothidea* cannot proliferate without larva	Camp (1981), Weis (1982), Heath and Stireman (2010), Janson et al. (2010)
Asphondyliini	*Asphondylia auripila* (group)	*Sphaeropsis* (*Botryosphaeria* teleomorph)	^c^	^c^	^c^	Nutrition, ^^c^^buffering larva from plant antiherbivore chemistry	Fungal mycelium forms a thick mat connected to the gall tissue by intercellular haustoria; larva rotates, feeding evenly on the substrate	Huggins (2008)
	*Asphondylia borrichiae*	*Alternaria alternata* ^b^, *Bipolaris* sp., *B. dothidea* ^b^, *Cladosporium* spp.^b^*, Sarocladium* sp.^b^, *Fusarium* ^b^ (21 genera)	Ovipositing female; ^c^horizontal; some of the fungi may preexist in plant tissue and expand with larval activity; parasitoids	Yes	Dispersal	Nutrition	Fungal isolates similar between host plant and gall tissues (horizontal transmission); fungal gall diversity is associated with the AGM–plant relationship	Rossi et al. (1992, 2006), Stiling et al. (1992), Te Strake et al.(2006), Rowan (2014), Sharpe (2024)
	*Asphondylia glabrigerminis*	*B. dothidea* ^b^ (*Dichomera* synapomorph)	Ovipositing female, proliferation of the spores deposited on gall surface; ^c^horizontal (environment)	Yes	^c^Proliferation	^c^Nutrition	Larva may regulate mycelial development and sporulation; ^^c^^interdependence	Adair et al. (2009)
	*Asphondylia floriformis, A. sarcocorniae, A. tecticorniae, A. peelei*	*B. dothidea* ^b^*/B. corticis* ^b^*/B. berengeriana* ^b^	^c^Horizontal (both host generalist and specialist species in all the lineages of Botryosphaeriaceae)	^c^No	^c^Dispersal	Nutrition; protection against parasitoids is unlikely	Lots of microfungi isolated from the upper surface of the gall, but only *B. dothidea* was present in larval chambers	Lebel et al. (2012), Rixon et al. (2021)
	*Asphondylia melanopus*	*Macrophomopsis coronillae* (*Botryosphaeria* teleomorph), *Cladosporium* sp.	^c^	Yes	^c^Living space	Nutrition	*M. coronillae* is associated with *A. melanopus*, while *Cladosporium* is a common endophyte secondarily colonising larval chamber	Kehr and Kost (1999)
	*Asphondylia nepetae*	*Cladosporium* spp.^b^, *Fusicoccum aesculi* (*B. dothidea* anamorph)^b^, *Epicoccum* ^b^, *Fusarium* ^b^, *Lecanicillium* ^b^, *Penicillium* ^b^	^c^	^c^	^c^; proliferation of *Cladosporium* independent of larva	^c^Nutrition; larva feeds upon plant material at early development stages	*Cladosporium* dominates and does not show any antagonistic relationships with *B. dothidea*	Bernardo et al. (2018)
	*Asphondylia prosopidis*	*B. dothidea* ^b^	^c^	^c^	^^c^^Mutualistic relationship	^c^Nutrition, mutualistic relationship; larvae may regulate the virulence of *B. dothidea*	Lack of genetic variation of these *B. dothidea* strains from USA compared with other *Asphondylia* species from South Africa	Park et al. (2019)
	*Asphondylia sarothamni*	*Macrophoma* (coelomycete anamorph of *Botryosphaeria*)	^c^Ovipositing female (conidia traces found in mycetangia)	Yes	^^c^^Living space, inquiline	Nutrition	Nutritive mycelium typical of mycetophages is present, and larval-induced nutritive tissue is absent	Ross (1932), Richter-Vollert (1964), Meyer (1^987^), Bronner (1992), Rohfritsh (2008)
	*Asphondylia serpylli*	*B. dothidea* ^b^ (anamorph *Fusicoccum aesculi*), *Cladosporium, Alternaria*	^c^; at least *Cladosporium* does not depend on inoculation by *Asphondylia* and its parasitoids	^c^	^^c^^	^^c^^Nutrition	Parasitoids may feed on fungal mycelium, after devouring the host	Zimowska et al. (2017)
	*Daphnephila sueyenae, D. stenocalia, D. ornithocephala, D. truncicola, D. taiwanensis*	*B. dothidea* (anamorphs *Fusicoccum aesculi* and *Dichomera saubinetii*), *Fusarium, Pestalotiopsis, Pestalotia*	^c^	^c^	^^c^^	^^c^^Larval development, nutrition (obligate)	Galls contain both nutritive tissues typical for non-ambrosia gall midges and fungal hyphae; mycophagy–phytophagy transition	Chao and Liao (2013), Pan et al. (2015)
	*Illiciomyia yukawai*	*B. dothidea* ^b^, *Phomopsis* sp.^b^, *Colletotrichum* sp.^b^, *Pestalotiopsis* sp.^b^	Horizontal (environmental source, not from the natal galls)	Yes	^c^	^^c^^Nutrition, protection of larva from attack by antagonistic fungi	*B. dothidea* in mycangia of adults in high frequency; seasonal changes of fungal composition in the galls	Kobune et al. (2012)
	*Schizomyia galiorum*	*Camarosporium macrosporium*	^c^Ovipositing female (source of conidia unknown)	^c^	Living space; ^^c^^inquiline	Gall initiation, larval development (obligate); nutrition	Larvae pierce the fungal cells, which maintains the mycelium as a thin, flat layer that may be mistaken for plant nutritive tissue (pseudo-parenchyma)	Rohfritsch (2008)
Lasiopterini	*Lasioptera arundinis*	*Ramichloridium subulatum, Macrophoma* (*Sphaeropsis; B. dothidea* anamorph); *Myrmecridium, Sporothrix, Radulidium, Cercospora beticola* species complex^b^	Horizontal, ovipositing female (conidia from decaying leaf sheaths of their host plant)	Yes	Dispersal	Nutrition (fungus + stem cells; fungus facilitates the stem penetration), protection against parasitoids, facilitation of the imago exit	Nutritive mycelium typical of mycetophages (larval-induced nutritive tissue is absent); fungus aids the larvae in feeding by forming connecting hyphae between them and the vascular bundles of the host	Rohfritsch (1992, 1997, 2008), Meyer (1987), Bronner (1992), Ryckegem (2005), Yukawa and Rohfritsch (2005), Pyszko et al. (2024)
	*Lasioptera donacis*	*Arthrinium arundinis, Sarocladium subulatum* ^b^, *Penicillium sumatrense* ^b^, *Galactomyces candidum, Fusarium* ^b^	^c^	^c^	Dispersal, plant colonization and proliferation (obligate)	Nutrition	*A. arundinis* requires larval feeding to grow and develop—fungus can only colonize parts damaged by larva	Goolsby et al. (2017), Bon et al. (2018, 2023)
	*Lasioptera rubi*	*Dothiorella* (*Botryosphaeria* teleomorph), *Fusarium avenaceum, Leptosphaeria coniothyrium, Didymella applanata*, or *Colletotrichum gloeosporioides*	^c^	Yes	Dispersal, inoculation, development and expansion inside the plant tissues	Nutrition, penetration of the plant tissue	Galls have big holes, irregular cavities and tunnels covered with mycelium with the larva feeding from it; interdependence (*Dothiorella*), other fungi may be opportunistic pathogens	Kaiser (1978), Tastás-Duque and Sylvén (1989), Tsolova et al. (2022), Popescu and Gostin (2024)

a Only AGM species with identified fungal symbionts from the gall interior and including sufficient additional information about modes of transmission, benefits of the interaction, etc., were included. Presumed obligate associations (from the insect, fungal, or both sides) are in bold, and fungal symbionts with proven obligate relationship are underlined.

b Detected by molecular identification.

c Lacking or possible (but not experimentally verified) information.

Based on our recent study (Pyszko et al. 2024), the galls of Asphondyliini and Lasiopterini harbour similar fungal communities dominated by *B. dothidea*, suggesting that the gall interior provides an environment that supports the growth of similar fungi, regardless of the host plant or evolutionary relationships among the AGM species. In Asphondyliini, a community consisting of one specific fungus supplemented with multiple nonspecific fungi may be common (Tokuda and Yukawa 2007, Raman and Suryanarayanan 2017). This pattern may have evolved over time; basal clades of Asphondyliini (e.g. stem-galling *Daphnephila* sp.) may be associated with multiple fungi, while more derived clades (e.g. leaf-galling *Daphnephila* species) may rely on specific fungus (Pan et al. 2015, Raman and Suryanarayanan 2017). This hypothesis remains to be tested in Lasiopterini. Nevertheless, the pattern of fungal communities may also have a macroecological explanation; the need for multiple fungal associates may have arisen in warm, humid habitats, where gall inducers may have acquired spores of various fungi from the environment (Pan et al. 2015), or a temporal explanation, where the number of fungal species in the galls increases as they develop (Kobune et al. 2012). Bissett and Borkent (1988) proposed that fewer fungal species than cecidomyiids may be involved in ambrosia galls, and that several cecidomyiids may use the same fungus (Adair et al. 2009), which aligns with the results of our recent study (Pyszko et al. 2024). Nevertheless, given the existence of contrasting theories and contradictory results, the specificity of the interaction between fungi and AGM remains unresolved.

Due to a lack of experimental evidence, fungi in AGM galls are commonly referred to as ‘associated’ rather than mutualists and sometimes debated as incidentally sampled saprophages due to methodological inconsistency and a lack of experimental design or intensive sampling (Batra and Lichtwardt 1963, Janson et al. 2010). Fungal associates have been studied mostly by cultivation and subsequent determination by taxonomists (e.g. Wilson 1995, Malagaris 2011, Pan et al. 2015, Zimowska et al. 2017) or standard barcoding on purified culture (e.g. Adair et al. 2009, Janson 2010, Kobune et al. 2012, Lawson et al. 2014, Heneberg et al. 2016, Bernardo et al. 2018, Bon et al. 2018, Nagle et al. 2019, Zimowska et al. 2020), or barcoding of raw samples (Park et al. 2019). Nevertheless, cultivation methods commonly provide incomplete information; in addition to capturing only a fragment of diversity due to the dominant species outcompeting less competitive species on the agar medium (Boddy and Hiscox 2016), they are prone to contamination (Amann et al. 1995), and frequently fail (e.g. Krischik et al. 1989, Adair et al. 2000), even for the dominant *B. dothidea*. This may be because this fungus may have lower ability to propagate in artificial media compared to the inner surface of the gall. Unlike its free-living variant, it also exhibits some physiological changes, possibly induced by biological factors such as AGM larval secretions (Heath and Stireman 2010, Kobune et al. 2012), further complicating its successful cultivation. Furthermore, the nomenclatural inconsistency within the *Botryosphaeria* species complex, which was recently resolved following the epitypification of *B. dothidea* (Slippers et al. 2004), may explain some reports referring to *Macrophoma, Diplodia*, and *Dothiorella* (Denman et al. 2000, Lebel et al. 2012, Zimowska et al. 2017, Park et al. 2019). Therefore, taxonomists recommend a careful interpretation of previous records of these fungi (Phillips et al. 2013, Marsberg et al. 2017).

The AGMs represent a complex system comprising the plant, gall, and larva inside the gall. Therefore, to fully understand the AGM microbiome, all the respective parts should be considered. However, such comprehensive analyses have only been performed occasionally (Pyszko et al. 2024). Except for internal surfaces, fungal samples were collected from the external surfaces of the galls (Nagle et al. 2019), flowers apparently not colonized by AGM (Bernardo et al. 2018), egg surface (Janson et al. 2010), and larval surface (Kobune et al. 2012). The latter study also mentioned the urgency for the detection of fungal DNA from larval gut, where the larval surface would be compared with its interior. However, this is technically very difficult, considering the small size of the larvae. Our recent study comparing the gall surface, nutritive mycelia, and entire larvae found high richness and taxonomic diversity in larval mycobiomes, distinct from the other parts (Pyszko et al. 2024), but the specificity and role of larvae-associated fungi remain unresolved.

Modern molecular analytical tools for identifying entire microbial community (next-generation sequencing) could provide a clearer picture of microbial diversity in the AGM galls. However, DNA metabarcoding alone is not sufficient to fully elucidate these complex interactions. The integration of metatranscriptomics and metabolomics can offer deeper insights into the functional dynamics and metabolic pathways of these communities. Advanced techniques such as fluorescence *in situ* hybridization can help unravel specific spatial distribution patterns of fungi within galls and larvae (Frickmann et al. 2017). Furthermore, using null models to assess the stochasticity of microbiota composition can improve our understanding of the ecological processes shaping these communities (Sloan et al. 2006). Controlled experiments that examine the effects of different environmental conditions on the structure and function of the microbial community are also crucial. Combining these approaches will facilitate a comprehensive understanding of microbial consortia and their influence on gall development and insect–fungi–plant interactions.

## Mutual benefits for insects and fungi in AGM galls: roles in nutrition, defence, and development

**Figure 2.**
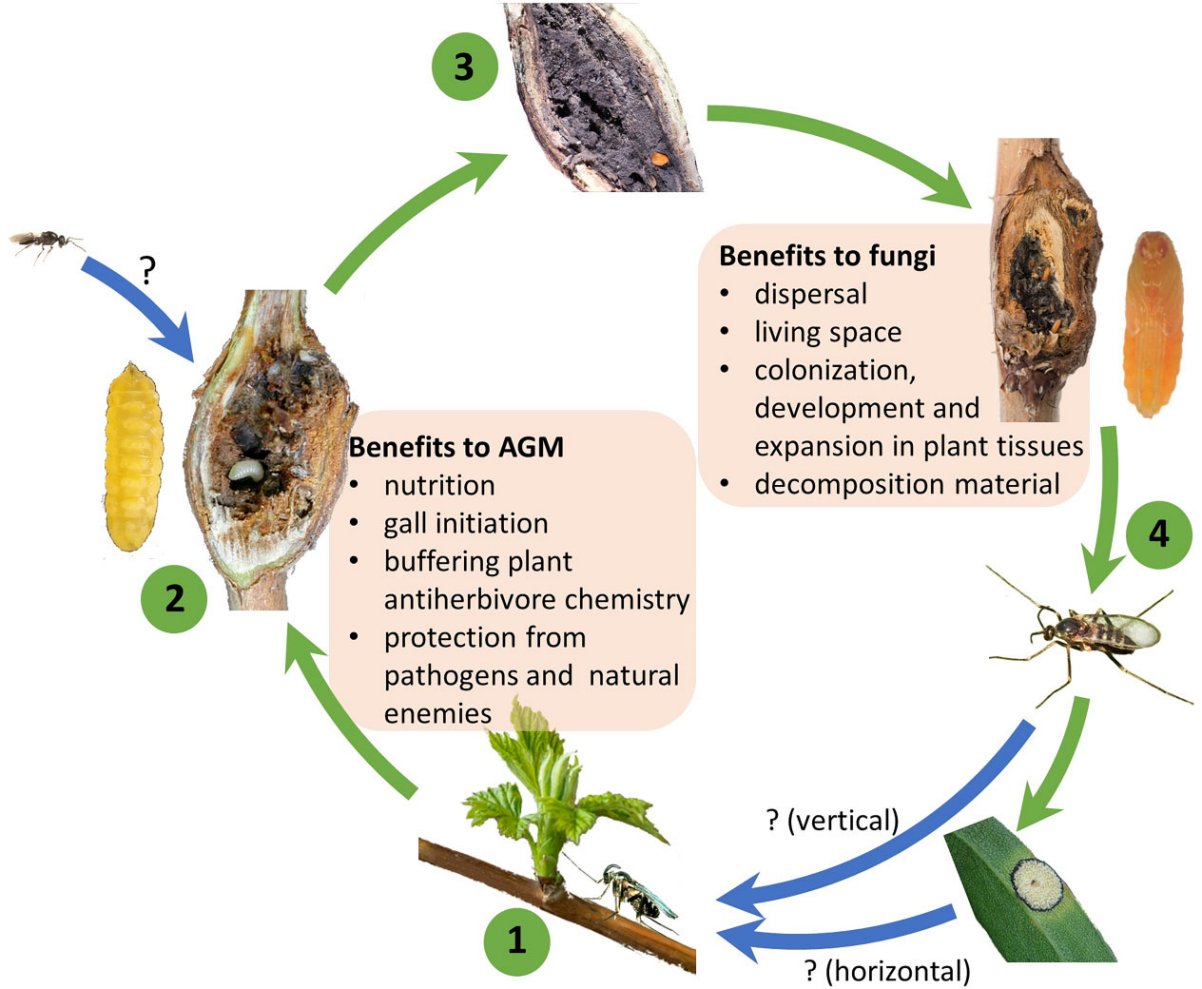
Illustrative life cycle of a typical AGM, showing possible benefits of its interaction with fungi (using *Lasioptera rubi*, its fungal symbiont, *Dothiorella* [*Botryosphaeria* teleomorph], and a parasitoid *Platygaster*, in the figures). A mated female lays eggs in plant tissue and inoculates fungal conidia into the wound, initiating gall formation and growth (1). Fungal hyphae proliferate inside the gall, eventually overgrowing the larva that feeds from it. Larvae are frequently attacked by hymenopteran parasitoids (2). The larva pupates inside the gall (3). After eclosion, the adult female mates and either searches directly for new host plant, carrying fungal conidia from its natal gall, or seeks fungal conidia in environment (4). Arrows styles or positions correspond to possible transmission routes of the fungus into the system. Photo of open gall (part 2) was adapted from ‘Plant Parasites of Europe’ (under a CC BY-NC-SA 4.0 license). Other photos of *L. rubi* galls, larva, pupa and adults were adapted from ‘artsdatabanken’ by Hallvard Elven (under a CC BY license). The photo of *Platygaster* was adapted with permission from ‘BugTracks’, copyright by Charlye Eisemann. The photo of *Botryosphaeria* was adapted from ‘CRO mushrooms’ by Zihao Wang (under a CC BY license).

Over time, perspectives have shifted from considering fungal associates as merely incidental inquilines (Ross 1932) to recognizing them as mutualists (Docters Van Leeuwen 1929, Docters van Leeuwen 1939). According to the current state of knowledge, fungi in AGM galls can serve two primary functions: (i) providing nutrition and (ii) secreting compounds that control gall growth (Bissett and Borkent 1988, Amann et al. 1995, Lawrey and Diederich 2003, Rohfritsch 2008, Adair et al. 2009). In return, AGM may facilitate fungal dispersal, provide a protected living space, and promote hyphal proliferation within the gall (Heath and Stireman 2010) (Table 1 and Fig. 2).

The extent to which AGM larvae feed on fungi in the galls remains unknown. Multiple studies have suggested that larvae may feed on fungus rather than on plant tissue (Bissett and Borkent 1988, Rohfritsch 2008, Adair et al. 2009). Conversely, some studies suggest that larvae previously identified as mycophagous may instead feed on plant tissues (Richter-Vollert 1964, Borkent and Bissett 1985, Gagné 1989, Herman et al. 1993, Yukawa and Rohfritsch 2005; Rohfritsch 2008; Adair et al. 2009, Stireman et al. 2010). However, after a long debate (Gagné 1968, Bissett and Borkent 1988, Gagné 1989), the nutritive role of fungi and plant in the AGM–fungi–plant system has so far been reliably resolved only in the genus *Asteromyia*; by *in vitro* culturing (Heath and Stireman 2010) and *in vivo* analysis of sterols (Janson et al. 2009), it has been demonstrated that this AGM feeds on *B. dothidea*. Several more or less successful attempts have been made in other AGM taxa to prove that larvae can thrive on a fungal diet *in vitro* (Kaiser 1978, Haridass 1987, Meyer 1987, Rowan 2014, Bernardo et al. 2018), with some larvae even appearing to form gall-like structures on agar plates (Heath 2012). Nevertheless, demonstrating fungal consumption *in vitro* is nearly impossible for most AGM species. The larval mortality rate is extremely high (Heath 2012); larvae commonly die after a few days of rearing, and are colonized by the underlying fungus, possibly due to the absence of the constraints on fungal growth found in the conditions of the gall interior, disrupting the natural interplay between the fungus, larva, and plant (Bernardo et al. 2018).

Therefore, a more feasible approach to investigating the extent of larval consumption of fungi is to analyse sterols within the complex system of an intact gall. Insects lack the ability to synthesize the sterols necessary for metabolic and structural purposes; therefore, they acquire these substances from their diet (Jing and Behmer 2020). The AGM larvae can utilize either plant phytosterols as precursors or fungal ergosterols. In *A. carbonifera*, fungal ergosterol metabolites have been detected instead of plant-derived phytosterols, demonstrating the consumption of the fungal ectosymbiont (Janson et al. 2009). In addition to sterols, it is hypothesized that fungi in ambrosia galls may provide AGM with vitamins and amino acids (Herman et al. 1993), which is a phenomenon known from ambrosia beetles (De Fine Licht and Biedermann 2012, Reverchon et al. 2021). Analysing the histochemical and cytological profiles of galls and their associated primary metabolites represents a promising direction of future research on the nutritional role of fungi in AGM galls. Different cellular pathways in gall tissues are activated depending on the feeding mode of the gall inducer and the involvement of fungal symbionts (Bragança et al. 2022).

An important function of fungi may be the induction of gall growth and morphogenesis (Stone and Schönrogge 2003) through the secretion of gall-inducing compounds, as shown for *Sclerotium asteris* in *Asteromyia carbonifera* galls (Camp 1981). However, mycelial proliferation may be delayed by several weeks compared to gall development, suggesting that gall growth is more likely induced by the larva (Bissett and Borkent 1988, Yukawa and Rohfritsch 2005). Furthermore, these contrasting theories stem from various observations; some studies have found fungal mycelium growing in the gall before the larva started feeding (Docters van Leeuwen 1939, Mani 1964, Nijveldt 1969), whereas others found the hyphae in the first developmental stages (Dorchin et al. 2002, Sá et al. 2009, Lebel et al. 2012, Rowan 2014). Conversely, other studies claim that the fungus may not be easily detected during the early stages, but as it enters the vascular tissue of the host plant, it is responsible for gall growth (Rohfritsch 2008), that first-instar cecidomyiid larvae occur without any signs of fungi but fungi appear in more advanced developmental stages (Adair et al. 2000), or that many ambrosia galls reach their full size before the fungi proliferate inside the gall (Dorchin et al. 2019). Either way, the larva affects fungal development through its digestive enzymes, and when it stops feeding, is attacked by a parasitoid, or dies, changes in the solidity and colour of the fungus are observed, at least in *Camarosporium macrosporium* in the galls of *Schizomyia galiorum* (Rohfritsch 1992, 2008), suggesting that the interaction between the fungus and the larva is dynamic and varies across different developmental stages and species.

The secondary functions of fungi may involve the protection of AGM larvae against natural enemies and other benefits. In some *Lasioptera* or *Asteromyia* species, the fungal mat (of their associates *Ramichloridium subulatum* and *B. dothidea*, respectively) may hinder the parasitoids in localizing the AGM larva (Abrahamson 1987, Rohfritsch 1992, Heath and Stireman 2010). It also provides mechanical protection against inquilines and parasitoids (possibly *Macrophoma* in the galls of *Asphondylia*, Sugiura et al. 2004; or *S. asteris* in *A. carbonifera*, Weis 1982). Further, endophytic fungi frequently produce alkaloids or other mycotoxins that deter or kill phytophagous insects, and herbivory can stimulate further production of these compounds (Hammon and Faeth 1993). In some galls, these fungi can render gall tissues unpalatable or even lethal to other insects, preventing secondary colonization (Wilson 1995). One of the best-known examples of an endophyte exhibiting such deterrence yet forming a mutualistic relationship is *Epichloë* sp. (Clay and Schardl 2002) allowing the mycophagous fly *Phorbia phrenione* to feed without apparent harm (Nyman and Julkunen-Tiitto 2000). Analogously, it is hypothesized that in AGM, the larvae-associated fungus—harmless to the midges themselves—may produce similar secondary metabolites, thereby providing a valuable layer of chemical protection for the gall (Sugiura et al. 2004, Yamazaki 2016). Fungi may also buffer larval penetration and progression within plant tissues and facilitate the exit of the imago (Rohfritsch 1992, 1997, Yukawa and Rohfritsch 2005, Rohfritsch 2008, Thomas and Goolsby 2015). In some fruit-dwelling *Asphondylia*, fungi are hypothesized to postpone fruit ripening, keeping it on the plant for longer (Krischik et al. 1989), which is similar to the production of green islands on leaves to extend their lifespan by certain leaf fungi (Kaiser et al. 2010). This mechanism may also protect larvae, as the green colour makes the fruit less attractive to frugivores (Highland 1964, Krischik et al. 1989), with the reduced frugivory being an unintended but beneficial side effect. In turn, insects may provide fungi with decomposable material, with larval frass serving as a supplemental nutrient source (Camp 1981), growing substrates well protected against unfavourable environmental conditions, dispersion (Sharma 1989, Bittleston et al. 2016), vectoring to favourable host plant (Bissett and Borkent 1988), spreading within plant tissues (Rohfritsch 1992), or inhibition of competing fungi (Bronner 1992, Rohfritsch 2008).

Furthermore, ambrosia galls have been reported to produce antibiotic compounds (Yamazoe et al. 2007). However, their exact origin remains unclear. Larvae can inhibit the growth of competing fungi through specific secretions (Bronner 1992, Rohfritsch 2008, Rowan 2014); in *A. carbonifera*, larval removal has been shown to affect fungal proliferation within the gall interior (Heath 2012). Nevertheless, nutritive mycelia themselves can inhibit the growth of competing fungi. The most frequently reported dominant fungus *B. dothidea* is frequently reported to produce metabolites with antimicrobial, antifungal, and cytotoxic activities (Xiao et al. 2014). Importantly, antibiotic-producing bacteria can also protect nutritive mycelia, much like they defend fungal gardens from antagonistic organisms in fungus-farming ants, bark beetles, and termites (Cafaro and Currie 2005, Currie et al. 2006, Scott et al. 2008, Cardoza et al. 2009, Barke et al. 2010, Um et al. 2013). Given the functional convergence of AGM fungal microbiomes with those of other fungus-growing insects (e.g. bark beetles; Stodůlková et al. 2015), symbionts are expected to outcompete harmful microbes without suppressing nutritional ones or affecting their hosts. Nevertheless, this interesting phenomenon remains largely unexplored in AGM and warrants further investigation.

## Fungal symbionts as drivers of species boundaries and diversification in AGM

In gall-forming Cecidomyiidae, the prevailing species concept based on host specificity can be misleading, particularly as insect–fungi associations may play a crucial role in host and interorgan alternations, diet breadth (Harris 1975, Joy 2013, Rowan 2014, Dorchin et al. 2015), host shifts, and adaptive radiation (Plakidas 1988, Parnell 2009, Tokuda 2012, Dorchin et al. 2015, Nagle et al. 2019). Certain fungal species may enable their AGM hosts to alternate between unrelated plant species and genera (Harris 1975). Although most AGM species are still considered as strictly monophagous and plant organ-specific (Hawkins et al. 1986, Gagné and Waring 1990, Joy and Crespi 2007, Raman 2007, Stokes et al. 2012, Dorchin et al. 2015), host alternation occurs disproportionately frequently in AGM compared to other gall-forming insects (Joy 2013). Some species are multivoltine, alternating between hosts available at different times of the year (Tokuda 2012), while others alternate between different organs of the same host plant (Plakidas 1988, Parnell 2009, Dorchin et al. 2015).

Fungal symbionts may confer the flexibility needed for such shifts (Bissett and Borkent 1988, Yukawa and Rohfritsch 2005, Uechi and Yukawa 2006, Nagle et al. 2019) by helping AGM overcome plant chemical defences or modifying defensive compounds (Huggins 2008). A similar process occurs in bark and ambrosia beetles, where symbiotic fungi degrade or detoxify toxic plant metabolites, enabling the beetles to overcome host chemical defences and colonize new plant species, allowing them to exploit a broader spectrum of hosts (De Fine Licht and Biedermann 2012, Chakraborty et al. 2020, Zaman et al. 2023). Indeed, recent studies on AGM have revealed that multiple supposedly monophagous *Asphondylia* species actually belong to a single polyphagous lineage developing on several host plants (Gagné and Orphanides 1992, Yukawa et al. 2003, Dorchin et al. 2015, Uechi et al. 2018, Gagné and Jaschhof 2021). However, this view is challenged by evidence that some AGM taxa previously considered polyphagous may actually consist of groups of monophagous cryptic species (Zachariades et al. 2011, Pan et al. 2015), with interorgan shifts potentially being influenced by their fungal symbionts promoting this speciation (Nijveldt and Yukawa 1982, Gagné 1989, Yukawa et al. 2005, Russo 2006, Skuhravá and Skuhravý 2007, Pan et al. 2015, Gagné and Jaschhof 2021). Since certain species attack multiple hosts within the same family but consistently target the same plant part, we hypothesize that plant organ specificity may be a stronger determinant of host utilization than plant species. This pattern parallels endophytic fungi, which often exhibit specificity toward particular plant organs (e.g. leaves or roots), suggesting that organ specificity may outweigh host species specificity in shaping insect–fungi interactions (Wearn et al. 2012). A good model group for testing this hypothesis can be Central European *Asphondylia* spp. that develop in Fabaceae. Most species form galls in pods, whereas a single species, *A. ononidis*, attacks primarily leaf axils (Skuhravá et al. 2008), suggesting that it could be a separate species. This reaffirms the urgent need for taxonomic revision of AGM species.

Although the exact causes of host range expansions remain unknown, oviposition mistakes or extreme shortage of host plants could serve as potential triggers (Yukawa et al. 2016, 2019), as was shown for *A. gennadii*, ovipositing and successfully developing in plants from several families in captivity (Orphanides 1975, Gagné and Woods 1988). However, such shifts may come at a lower cost due to fungal symbionts, which can help mitigate plant defence barriers, enabling larvae to survive and develop on suboptimal or novel hosts. As evidenced by other insect–plant systems, lowered physiological costs of host switching can foster more frequent colonization of new or marginal hosts, ultimately contributing to pest status (Moise et al. 2023). Consequently, several AGM species are serious pests attacking important agricultural plants, with multiple lines of evidence indicating recent shifts, especially on introduced plants (e.g. Gagné and Woods 1988, Perdikis et al. 2011, Uechi et al. 2018, Yukawa et al. 2020). Host–plant shifts may also result from a high pressure of parasitoids and predators, as parasitism rates in AGM are extremely high and possibly linked to their fungal associates. For instance, fungal symbionts might alter gall morphology or chemistry in ways that attract parasitoids, potentially shaping the balance between the costs and benefits of shifting to a new host (see the ‘Role of fungi in tetratrophic interactions’ section). This shift would be in line with the enemy-free space hypothesis, as evidenced in the dipteran leaf miner (Gratton and Welter 1999). However, such a link has never been studied in the AGM. Investigating the ratio of galled host plant parts to the available parts versus the proportion of parasitized galls relative to galled plant parts in polyphagous AGM species across different host plants, alongside characterizing the fungal communities and their transcriptional activity in each scenario, could help reveal the role of parasitoids and fungal symbionts in the host shift of AGM by identifying symbiont-derived metabolic pathways involved in this tetratrophic interaction.

Thus, AGM may serve as excellent models for studying the significance and evolutionary context of host shifts, with fungal symbionts playing a pivotal role in these processes. Further studies combining extensive observations with the molecular and functional identification of fungal symbionts are necessary to elucidate the role of fungi in presumed host- and plant-part-related shifts. Understanding host specialization in gall-forming insects associated with fungi requires knowledge of both their phylogenetic ancestry and diet. Given that shifts from the original plant part or host, facilitated by fungal partners, may have occurred during evolution, it is necessary to reconstruct the phylogeny of the group, inspect the specialization of the sister groups, and infer the potential scenarios for AGM evolution.

## Potential routes of symbiont transmission

Understanding the origin of AGM–fungi interactions cannot be solved without understanding where AGM obtain their fungal symbionts. Despite advances in molecular methods facilitating the detection of fungal traces, the sources of fungi and the exact mechanisms of their acquisition and transport remain poorly understood (Gagné 1989, Rohfritsch 2008, Heath and Stireman 2010). Fungal symbionts can be transmitted vertically, with the female acquiring the fungus before or during emergence, then carrying it to the new oviposition site and passing it to the offspring along with the egg deposition, as seen in ambrosia beetles (Janson et al. 2010, Li et al. 2019). Alternatively, they can be acquired by the ovipositing female from the environment each generation through horizontal transfer (Fig. 2), as seen in termites and woodwasps (Aanen et al. 2007, Pažoutová et al. 2010). Vertical transmission is often indicated by the presence of the specialized spore-carrying organs, such as mycetangia (Biedermann and Vega 2020), although these same structures may also facilitate fungal collection from environment in some AGM species. Anyway, their presence in some AGM females (see Table 1) suggests their ability to carry fungal conidia and to transfer and inoculate them into the host plant along with the eggs during oviposition (Docters Van Leeuwen 1929, Skuhravá and Skuhravý 1981, Borkent and Bissett 1985, Abrahamson 1987, Bissett and Borkent 1988, Gagné 1989, Bronner 1992, Dreger-Jauffret 1992, Yukawa and Rohfritsch 2005, Rohfritsch 2008, Heath and Stireman 2010, Lebel et al. 2012).

**Figure 3.**
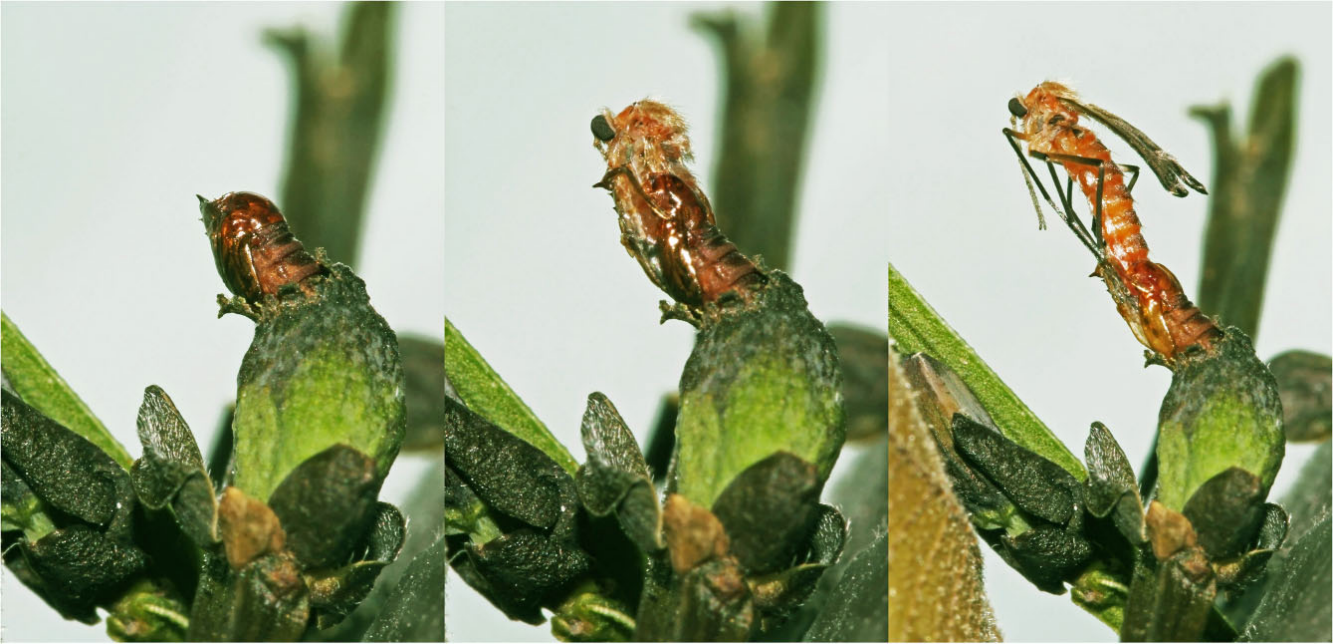
The final ecdysis of *Asphondylia sarothamni* (Rübsaamen, 1917) occurs externally (photo by Jan Ševčík).

However, previous studies have failed to reliably determine the origin of conidia (Janson et al. 2010, Park et al. 2019). Despite occasional reports of conidial transfer from galls during female emergence (Skuhravá and Skuhravý 1981), most studies support active acquisition of conidia from unknown environmental sources (Bissett and Borkent 1988, Tastás-Duque and Sylvén 1989, Rohfritsch 2008, Adair et al. 2009, Janson et al. 2010, Kobune et al. 2012). This may be due to the fact that freshly emerged females often do not carry conidia in their mycetangia (Bissett and Borkent 1988, Rohfritsch 2008, Adair et al. 2009, Janson et al. 2010, Kobune et al. 2012), probably because the final ecdysis occurs externally (Adair et al. 2009) (Fig. 3). However, conidia have been detected in the mycetangia of ovipositing females (Bissett and Borkent 1988, Rossi et al. 1999, Te Strake et al. 2006, Rohfritsch 2008, Adair et al. 2009, Janson 2010, Kobune et al. 2012), and analyses of their shape suggest a biotrophic source (Heath 2012). This suggests that the morphology of conidia is consistent with fungi that thrive on living tissue. This supports the idea that the fungus–AGM relationship involves a fungus that grows in the inner part of the gall, rather than externally on dead plant or decaying material. However, most studies investigating the presence of fungi in mycetangia of freshly emerged females (Bissett and Borkent 1988, Rohfritsch 2008, Heath and Stireman 2010) were performed many years ago, without the access to modern analytical tools, and at least one report contradicted the theory of conidia origin from galls (Borkent and Bissett 1985).

Similarly, the exact mechanisms of identifying specific fungal conidia, their potential collection, and transport from the environment remain poorly understood (Borkent and Bissett 1985, Gagné 1989, Yukawa and Rohfritsch 2005, Te Strake et al. 2006, Rohfritsch 2008, Rowan 2014). Females may distinguish among different fungi present in the environment, probably not only by olfaction, but also visually by the shape and size of conidia (Rohfritsch 1997). Despite reports suggesting the presence of fungal spores in the depression of the ovipositor (Veenstra et al. 2019), the hypothesis that females actively collect conidia in specialized structures on their ovipositors has not been explicitly tested (Dorchin et al. 2015). Heath and Stireman (2010) observed females of *A. carbonifera* touching or dragging their ovipositor on a leaf surface, which may be a behaviour associated with the collection of fungal conidia (Veenstra et al. 2019). Despite evidence of their biotrophic origin, conidia may also be collected from decaying leaf sheaths (Herman et al. 1993; Rohfritsch 1997, 2008), dead twigs, stem cankers, and incomplete galls where midge or parasitoids have died (Adair et al. 2009), fruiting fungus found on old non-natal galls in the leaf litter (Bissett and Borkent 1988), and other parts of the host plant (Janson et al. 2010). The hypothesis of faecal spore transfer (Haridass 1987) remains untested and requires investigation. This point is underscored by the observation of miscellaneous detritus during a direct survey of mycetangia (Borkent and Bissett 1985). However, attempts to identify the exact sources failed due to the inability of AGM to initiate galls in enclosures (Rossi et al. 1999, Heath and Stireman 2010).

Fungal symbionts can be vertically transmitted, at least in the genus *Asphondylia* (Meyer 1952, Gagné 1989, Kolesik et al. 1997). The presence of unique clonal strains shared between certain *Asphondylia* species suggests limited genetic variation within *B. dothidea* (Park et al. 2019), reinforcing the likelihood of vertical transmission. However, genetic variation appears to be associated with geographic distance, rather than AGM species differences (Marsberg et al. 2017, Zimowska et al. 2020), suggesting the possibility of horizontal transmission. Examining a different AGM system may help clarify this issue. In the association of *A. carbonifera–B. dothidea*, the midge also appears to be associated with a single fungal lineage as in the case of unique clonal strains in *Asphonydylia* (Janson et al. 2010). This symbiont thus exhibits a degree of specificity that would not typically be expected if horizontally acquired acquisition (Adair et al. 2009), because non-endosymbiotic associations are particularly susceptible to invasion and symbiont replacement (Buchner 1965, Janson et al. 2010). However, the symbiotic *B. dothidea* in *A. carbonifera* is genetically identical to free-living populations (Janson et al. 2010). Furthermore, *B. dothidea* does not exhibit molecular evolutionary patterns typical of obligatory microbial symbioses, such as substitution rate acceleration or A+T nucleotide bias (Janson 2010), further supporting its horizontal transmission.

However, previous studies on the origin of symbiotic *B. dothidea* in ambrosia galls relied on amplified fragment length polymorphism (AFLP) genotyping, a method with several limitations: possible non-homology of comigrating fragments belonging to different loci, need for subjectively determined criteria for acceptance of bands in the analysis, non-independency of AFLP bands, and suboptimal reproducibility (Savelkoul et al. 1999, Meudt and Clarke 2007). To obtain more reliable data on symbiont transmission, advanced methods based on next-generation sequencing (e.g. microsatellite markers; Manawasinghe et al. 2018) should be employed.

If we assume that *B. dothidea* is transmitted horizontally, the question remains: how can such a specific host–symbiont mutualism be stabilized without vertical transmission? Nevertheless, this is possible; as a parallel association can be found in Plataspidae (Hemiptera), where *Riptortus pedestris* acquires a specific bacterial symbiont (*Burkholderia*) from the soil each generation (Kikuchi et al. 2007, Takeshita and Kikuchi 2017). Experimental inoculation of insect hosts with a broad range of bacterial taxa has revealed that microbial competition plays a crucial role in maintaining symbiotic specificity through competition-based selection. While a variety of bacterial taxa can colonize the gut and provide beneficial effects, co-inoculation consistently leads to the outcompetition of less competitive bacterial taxa in the harsh conditions of the gut. As a result, hosts provide a specific environment that selectively cultivates favourable symbionts and maintain symbiont quality (Itoh et al. 2019). Furthermore, AGM larvae have the ability to impact the pathogenic fungus *B. dothidea* by establishing specific conditions that promote its dominance over other fungal species. This selective environment not only ensures the presence of *B. dothidea* but also regulates its metabolism, thereby suppressing its pathogenicity (Raman and Suryanarayanan 2017, Park et al. 2019). The intricate interaction between AGM larvae and metabolically modified *B. dothidea* generates a distinct ecological niche, potentially influencing the colonization of other organisms within the galls. These findings underscore the need to study the entire AGM system, including both the galls and the larvae.

## Evolutionary pathways of the AGM–fungi interaction

The transition from mycophagy, which is an ancestral feeding type in Diptera (Labandeira et al. 2005), to gall-forming herbivory in Cecidomyiidae was either with AGM as a transitional stage, or direct (with AGM having arisen as secondarily mycophagous species). Phylogenetic, anatomical, and ecological analyses suggest that both routes have arisen multiple times (Dorchin et al. 2019). An example of an indirect transition is seen in the two evolutionarily distinct tribes, Lasiopterini and Asphondyliini, which diverged from Oligotrophini and Cecidomyiini, respectively (see Fig. 1), during their adaptation from mycophagy to a mixed type of diet (phytomycetophagy), reflecting ancestral associations with fungi, but still not becoming typical phytophages (Mamaev 1975, Bissett and Borkent 1988, Rohfritsch 1992, Roskam 2005).

On the contrary, some advanced midges from the supertribe Cecidomyiidi have secondarily ceased gall formation and become mycophagous, feeding on fungal fruiting bodies (e.g. *Camptodiplosis* Kieffer, 1913, tribe Mycodiplosini) (Nijveldt 1969), or various moulds growing on fruiting bodies (*Mycodiplosis* Rübsaamen, 1895 and *Karshomyia* Felt, 1908) (Henk et al. 2011, Fedotova and Perkovsky 2019). The larvae of some *Mycodiplosis* species feed on spores and mycelium of rust fungi (Pucciniales), benefiting infected plants by reducing the quantity of fungal spores through grazing (Golenia 1961, Kaushal et al. 2014). Conversely, adults may serve as interplant fungal vectors (Kushalappa and Eskes 1989, Stephanie et al. 2001). A similar coevolutionary process may have shaped fungal associations in ambrosia galls, potentially resulting from the opportunistic colonization of pathogenic fungi within galls or the exploitation of endophytic fungi as a food source or oviposition substrate (Stireman et al. 2010).

In this context, the interaction between AGM and endophytes appears to be crucial. Herbivorous insects encounter endophytes through contact with their host plants, where fungi are largely antagonistic (Carroll 1988, Clay 1989, Hata and Futai 1995, Wilson 1995). For instance, the abundance of cynipid galls (Hymenoptera) is positively correlated with tannin levels, partly because tannins protect larvae from endophytes (Taper and Case 1987). Cynipids even sequester tannins in gall tissues to a level of up to 60% (Russo 1983, Taper et al. 1986). Some endophytes can tolerate concentrations of tannins lethal for most fungi, but their growth can be retarded (Hammon and Faeth 1993). AGM can oppositely facilitate colonization of the galls by endophytes, and thus the endophytic mycobiota differs between infested and healthy plant tissues (Carroll 1986, Petrini et al. 1989). Te Strake et al. (2006) suggested that in *Asphondylia borrichiae*, associated obligatory fungi may either preexist in plant tissues or may be inoculated by the adult and invade surrounding plant tissues, similarly to mycophagous flies (Bultman and White 1988, Hammon and Faeth 1993). Nevertheless, the exact role of endophytes in the interaction between AGM and fungi has not been explicitly studied.

Given the unclear distinction between ambrosia and non-AGM species and their evolutionary trajectories, adult morphology and behaviour should be considered. Specialized conidia-carrying structures differ morphologically among individual tribes and are apparently not derived from homologous structures (Borkent and Bissett 1985). Ventral pouches (mycetangia) were found in some genera of Asphondyliini that may not have a symbiotic fungal association, even with fungal spores inside (Gagné 1989, Graham 1995). Well-developed but empty conidial pockets were found in several other genera and poorly developed or absent in some others (Borkent and Bissett 1985), which also suggests a gradient of symbiotically transitional stages of fungi utilization. If fungal symbiosis in AGM is a dynamic process, symbiotically transitional lineages likely exist at different stages of symbiont acquisition, integration, or loss of fungal symbionts (Stireman et al. 2010). Current evidence for evolutionary intermediate stages suggests transitions either between mycetophagy and gall formation or between (phyto)mycetophagy and phytophagy. A possible example is *Lasioptera berlesiana*, which feeds by piercing the mycelium of the phytopathogenic fungus *Sphaeropsis dalmatica* (an anamorph of *B. dothidea*) within the burrows created by *Bactrocera oleae* in olive fruits (Solinas 2011). Interestingly, *L. berlesiana* has also been hypothesized to transmit its inoculum to olive fruits (Moral et al. 2010). The latter case may be exemplified by galls of the genus *Daphnephila* Kieffer, 1905 on *Machilus zuihoensis*, which contain both nutritive tissues typical for non-AGM and fungal hyphae (Chao and Liao 2013).

Understanding AGM mating behaviour with respect to fungal utilization is essential but remains largely unexplored. Males and females can aggregate at conidia collection sites, and females carrying conidia may become more attractive. The utilization of different fungi across populations may eventually drive speciation, as behavioural modifications can provide sufficient reproductive isolation (Heath 2012). However, such hypothetical reproductive isolation would require divergence in fungal symbiont use, which seems highly unlikely given that in some Asphondyliini, the primary fungal symbiont appears to be almost exclusively *B. dothidea*. Nevertheless, this remains possible; despite the dominance of *B. dothidea*, the composition of nutritive mycelia is species-specific (Pyszko et al. 2024) and remains largely unknown. Further investigation into the role of adult preferences in fungal utilization may provide valuable insights into the evolutionary pathways leading to phytomycetophagy and AGM speciation.

## Role of fungi in tetratrophic interactions

The parasitism rate in ambrosia galls is unusually high, as they are heavily attacked by inquilines, primarily other cecidomyids (Dorchin et al. 2015, Yukawa et al. 2020), as well as thrips or mites (Wiesenborn 2015). Galls are frequently targeted by hymenopteran parasitoids, with parasitism rates in *Asphondylia* ranging from 30% to, most commonly, 80%–100%. The traditional hypothesis suggests that gall makers are less exposed to parasites, parasitoids, and predators than other insects due to the protective layer of plant tissue (Price and Clancy 1986, Price and Pschorn-Walcher 1988). However, the conspicuous appearance of the gall and the lack of mobility may be detrimental for gall formers in the presence of specialized parasites (Rossi et al. 1992, Clouse 1995). Indeed, among insect herbivores, parasitism rates increase with the degree of host concealment, reaching their highest levels in galling insects (Hawkins 1994). Yet, AGM may be protected by the fungal mat, at least in some *Lasioptera* or *Asteromyia* species (Abrahamson 1987, Rohfritsch 1992, Heath and Stireman 2010). Nevertheless, some parasitoids may feed on fungi or a combination of fungi and gall tissue after devouring their host (Waloff 1968, Parnell 2009, Zimowska et al. 2017). The hypothesis that fungal mats primarily protect larvae is further challenged by observations of very high parasitism rates (up to 80%) in certain *Asphondylia* species with thick fungal layers, suggesting that larval protection may instead depend more on gall structure and plant tissue thickness than on the presence of fungal mat alone (Lebel et al. 2012).

AGM larvae typically biosynthesize carotenoids (Heath and Stireman 2010), which can be degraded into volatile apocarotenoids by fungi (Zorn et al. 2003). Hymenopteran parasitoids use apocarotenoids as semiochemicals to detect their hosts (Zorn et al. 2003, Lewinsohn et al. 2005). Therefore, we hypothesize that the parasitism risk may be correlated with carotenoid levels in larvae. The presence of carotenoids has been confirmed in AGM galls; they are deposited with eggs and fungal spores through female accessory glands (Heath and Stireman 2010). They are also present in AGM larval salivary glands, the primary source of chemical agents that induce gall formation in cecidomyiids (Raman et al. 2005). AGM larvae may regulate mycelial growth, with sporulation and development of conidia occurring only when larvae fail to complete their development after being parasitized (Adair et al. 2009). Consequently, incomplete galls containing dead AGM larvae may serve as potential sources of conidia for adult AGM females, facilitating their dispersal (Bissett and Borkent 1988, Rohfritsch 1992, 1997, Adair et al. 2009, Bittleston et al. 2016). Therefore, fungal symbionts may act as antagonists to larval development. We hypothesize that fungi may attract parasitoids to facilitate their own reproduction, while simultaneously maintaining symbiosis with AGM by providing a source of conidia to females. Thus, fungi in AGM galls may play a crucial role in mediating fitness trade-offs between parasitoid attack and plant resistance (Heath 2012), though this theory warrants further validation.

## Concluding remarks

The continuum between taxa with a pronounced, well-developed fungal mat inside the gall and those lacking apparent mycelia (Richter-Vollert 1964, Gagné 1989, Yukawa and Rohfritsch 2005, Rohfritsch 2008, Kobune et al. 2012) makes the delimitation of AGM challenging. To fully capture the entire diversity of this group, species of supposedly non-AGM from the mentioned tribes must be examined for fungal presence and its role. Advanced next-generation sequencing methods could help address these issues—especially by inspecting taxa lacking apparent mycelia inside the galls—and elucidate the diversity and specificity of AGM-associated fungal communities. For instance, ongoing large-scale NGS surveys may soon provide valuable insights and expand our understanding of these associations. Furthermore, numerous Cecidomyiidae taxa require taxonomic revision, as the historically used species concept based on gall morphology and host specificity can be misleading (Dorchin et al. 2019, Kjærandsen 2022).

Additionally, investigating the mechanisms of horizontal and vertical transmission of fungal symbionts will be crucial for elucidating their ecological roles and evolutionary significance. The exact role of fungi in AGM fungal gardens remains unclear. Potential methods for evaluating their nutritional contributions include comparisons of ergosterol/phytosterol and isotope ratio analyses (15N/14N and 13C/12C), which can differentiate between plant and fungus dietary sources. Mating behaviour, conidia collection by adult AGM, and the origin of fungal symbionts are all areas requiring further exploration. Moreover, the interaction between AGM larvae and fungal partners in gall development and nutrition, along with the role of fungi in relation to natural enemies, warrants a detailed investigation. Finally, the specificity of fungus–AGM interactions and the role of bacterial symbionts in fungal gardens remain poorly understood. With their complex symbiotic relationships, AGMs offer a unique model for studying a range of evolutionary phenomena, providing valuable insights into insect–fungus coevolution and mutualism.
